# A pH-sensitive nanocarrier based on BSA-stabilized graphene-chitosan nanocomposite for sustained and prolonged release of anticancer agents

**DOI:** 10.1038/s41598-021-97081-1

**Published:** 2021-08-31

**Authors:** Sahar Gooneh-Farahani, Seyed Morteza Naghib, M. Reza Naimi-Jamal, Amir Seyfoori

**Affiliations:** 1 Research Laboratory of Green Organic Synthesis and Polymers, Chemistry Department, Iran University of Science and Technology (IUST), Tehran, Iran; 2 Nanotechnology Department, School of New Technologies, Iran University of Science and Technology (IUST), Tehran, Iran; 3 Biomaterials and Tissue Engineering Department, Breast Cancer Research Center, Motamed Cancer Institute, ACECR, Tehran, Iran

**Keywords:** Breast cancer, Biochemistry

## Abstract

Smart nanomaterials with stimuli-responsive behavior are considered as promising platform for various drug delivery applications. Regarding their specific conditions, such as acidic pH, drug carriers to treatment of tumor microenvironment need some criteria to enhance drug delivery efficiency. In this study, for the first time, pH-sensitive BSA-stabilized graphene (BSG)/chitosan nanocomposites were synthesized through electrostatic interactions between the positively charged chitosan nanoparticles and negatively charged BSG and used for Doxorubicin (DOX) encapsulation as a general anticancer drug. Physicochemical characterization of the nanocomposites with different concentrations of BSG (0.5, 2, and 5wt%) showed effective decoration of chitosan nanoparticles on BSG. Comparing DOX release behavior from the nanocomposites and free BSG-chitosan nanoparticles were evaluated at two pHs of 7.4 and 4.5 in 28 days. It was shown that the presence of BSG significantly reduced the burst release observed in chitosan nanoparticles. The nanocomposite of 2wt% BSG was selected as the optimal nanocomposite with a release of 84% in 28 days and with the most uniform release in 24 h. Furthermore, the fitting of release data with four models including zero-order, first-order, Higuchi, and Korsmeyer-Peppas indicated that the addition of BSG changed the release mechanism of the drug, enabling uniform release for the optimal nanocomposite in first 24 h, compared to that for pure chitosan nanoparticles. This behavior was proved using metabolic activity assay of the SKBR-3 breast cancer cell spheroids exposed to DOX release supernatant at different time intervals. It was also demonstrated that DOX released from the nanocomposite had a significant effect on the suppression of cancer cell proliferation at acidic pH.

## Introduction

Carbon nanostructures have attracted much attention in biomedicine due to their unique characteristics such as excellent mechanical^1–4^ and electronic properties^5–8^ as well as tunable flexibility and Young’s modulus^9,10^. Graphene, as one of the most functional two-dimensional (2D) carbon nanostructures, is a superb material with outstanding features used in various biomedical fields from biosensors to drug delivery^11–15^. Despite the unique properties of carbon-based materials such as graphene, concerns in the toxicity of these materials continue to challenge their usage in in-vivo bioengineering applications, including drug delivery formulations^16^. Herewith, the toxicity of carbon-based nanomaterials and particularly graphene nanosheets have been evaluated in a variety of studies so that the results have shown the dose-dependent toxicity of these nanomaterials^16–19^.

The non-specificity of the systemic chemotherapy methods causes several adverse effects on the healthy tissue following intravenous injection^20,21^. Also, repeated injections because of infusion pains, injection-related infections, as well as hospitalization of the sick cause the patient's unhappiness^22^. Therefore, the nano-formulation of drug delivery systems with the smart and prolonged anticancer drug release characteristics by reducing the harmful side effects and the number of injections leads to an improvement in the treatment efficiency and life of the patient^21,23–25^. The pathophysiological difference between the normal and the tumor tissues can be employed for the targeted and smart drug release to cancer cells. Due to glucose conversion to lactate in a tumor, the tumor tissue has a more acidic pH than the normal tissue^26,27^. Therefore, the systems that use pH-sensitive release are critical when they come to treat cancer. Additionally, during angiogenesis in the tumor tissue, irregular and abnormal blood vessel architecture causes the enhanced penetration and retention of the nano-carriers (EPR phenomenon)^28,29^. As a result, in the context of cancer chemotherapy, nano-size carriers with pH-sensitive properties with the capability of responding to the lower pH of the tumor microenvironment, are considered as a promising candidate to control the cancer therapeutics release rate in the peri-tumoral area. Therefore, nano-hybrid structures with pH-responsive properties can be potentially used to fulfill both requirements of nano-carrier formulation for great drug loading capacity, as well as smart drug release in chemotherapy strategies^30–32^.

Among the different materials used to prepare pH-responsive nano-carriers for smart drug release usage, chitosan-tripolyphosphate nanoparticles have attracted enormous interest, respecting their ease of preparation, cytocompatibility, and abundant chemical functionality^33–36^. However, despite the outstanding properties of these nanoparticles, their drug release behavior usually shows an initial burst release, in which much of the encapsulated drug tend to release at early times^37^. According to the literature, the burst release mostly depends on the physicochemical nature of the drug carrier and the affinities of the drug with the carrier^38^. For example, the physical loading of the drug and the instability of the drug carriers in the bloodstream, results in the rapid release and consequently uncontrolled high concentration of the drug over a short time. Therefore, sustained release is a prominent characteristic that ensures the physiological level of the drug to be durable and long-lasting under these conditions^38–40^. In this way, Mahmood et al. prepared chitosan-tripolyphosphate nanoparticles for the sustained release of docetaxel by modifications of the Calvo ionic-gelation method. The results showed faster release in the early hours of the nanoparticle administration, followed by the sustained release in just 24 h at pH 7.4^41^. The release curve exhibited that 60% of indole-3-acetic acid was released in the first 8 h. Afterward, the sustained release within 24 h leads to the release of 100% indole-3-acetic acid^42^.

The purpose of the present work is to introduce a simple and inexpensive method for synthesizing new stimuli-responsive nano-carriers with a prolonged and sustained release in which, not only early-stage burst release is inhibited, but also, continuous drug supply over a period of time is provided. Generally, the major loss of the drug depots in the early stage of the administration is a common shortcoming of the conventional stimuli-sensitive carriers, and the current study is going to address the current challenge. We suggested that adding graphene as the reinforcement in a low amount is a suitable method to provide sustained release. We first synthesized a new BSG/chitosan nanocomposite through electrostatic interactions between chitosan nanoparticles and BSG in three concentrations of BSG. Then, by evaluating the release curves in different pHs and their toxicity, 2 wt% BSG was selected as optimum. Additionally, the release mechanism of the optimal nanocomposite and its difference with the release mechanism of chitosan nanoparticles were investigated. Finally, the effect of optimal nanocomposite treatment on SKBR-3 breast cancer cells in 2D and 3D cultures, was tested to compare the in vitro results with in vivo model results. The results showed a controlled release with no burst effect in the early stages, even in acidic conditions for the optimal nanocomposite composition, so that the new nanocomposite could be introduced as a tremendous potential nano-carrier for cancer treatment.

## Materials and methods

### Materials

Chitosan with a molecular weight of 100,000–300,000 g/mol and tripolyphosphate used for the synthesis of chitosan nanoparticles were obtained from ACROS Organics company. Doxorubicin (DOX) as a model of the anticancer drug was prepared from EBEWE Pharma Ges.m.b.H. Nfg. KG company. Graphite, bovine serum albumin (BSA), and all other materials used to adjust pH and prepare a buffer were purchased from Merck company.

### Characterization

Morphology and shape of DOX-encapsulated chitosan nanoparticles and BSG/chitosan nanocomposites were characterized using MIRA3 FESEM of TESCAN company. Zeta potential of chitosan nanoparticles, DOX-encapsulated chitosan nanoparticles, BSG-chitosan nanocomposites dispersed in distilled water, and BSG dispersed in distilled water at pH 10, was measured using SZ-100z Zeta potential analyzer of Horiba Jobin Jyovin company. Size of chitosan nanoparticles, DOX-encapsulated chitosan nanoparticles, BSG-chitosan nanocomposites and BSG nanosheets dispersed in distilled water was obtained by SZ-100z Dynamic Light Scattering of Horiba Jobin Jyovin company. The synthesized BSG was confirmed by a UV–vis spectroscopy of T80+, and its thickness was evaluated by atomic force microscopy (AFM). FTIR spectra of chitosan nanoparticles and DOX-encapsulated chitosan nanoparticles were prepared in the wavenumber range of 500–4500 cm^−1^ using an FTIR-8400S spectrometer of Shimadzu company. Samples were mixed with KBr powder for pellets formation. Energy dispersive analysis of X-ray (EDAX) was also used to characterize the synthesized BSG-chitosan nanocomposites (Oxford Instruments X-MAX-80).

### Preparation of DOX-encapsulated chitosan nanoparticles

To prepare the chitosan nanoparticles loaded with DOX, we performed this: 0.5 mg/ml chitosan solution was prepared by dissolving 10 mg of chitosan in acetic acid (0.2 mg/ml) and stirred for 24 h. Then chitosan solution pH was adjusted to between 4.7 and 4.8 and filtering with a 0.45 μm syringe filter in the next steps. 0.5 ml of DOX (1 mg/ml) was added to 6 ml of a 0.5 mg/ml tripolyphosphate solution, pre-filtered with a 0.22 μm syringe filter, and stirred for 24 h on a magnetic stirrer. Finally, for the synthesis of nanoparticles, 6 ml of tripolyphosphate solution containing a drug cooled to 4–5 °C into 20 ml of chitosan solution was placed in a 60 °C water bath for 10 min, added dropwise and slowly and stirred for 10 min. The resulting nanoparticles were collected by centrifugation at 10,000 rpm within 10 min.

### Preparation of BSG

22 mg of BSA was dissolved in 400 ml distilled water and placed in an oven at 30° C for 14 h. Then, the pH of the solution was adjusted to 3.6 with 1 M hydrochloric acid. 0.6 g of graphite was added to the BSA solution, and the solution was placed under ultrasonic probing with a power of 15 W for 3 h at 25 °C or less to maintain the properties of the albumin structure. To precipitate and remove the remaining graphite and heavy several-layers BSG, the solution remains at ambient temperature for 24 h. Then, the supernatant was centrifuged for 10 min at a speed of 2000 rpm. The centrifuged supernatant contains either single-layer or few-layers graphene, collected with a high-speed centrifuge and then dried at ambient temperature^43^.

### Preparation of BSG-chitosan nanocomposites

BSG-chitosan nanocomposite was prepared in three different weight percentages (0.5, 2, and 5wt% BSG). To prepare nanocomposite, the following was done:

BSG was added to 20 ml of distilled water with pH 10 and placed under an ultrasonic probe within 30 min at 25 °C for uniform dispersion. Then, DOX-encapsulated chitosan nanoparticles were added and stirred at room temperature on a magnetic stirrer for 24 h. Finally, the precipitated solution was centrifuged and washed three times with distilled water, followed by drying at ambient temperature. The optimal percentage of BSG was selected based on the drug release study of three different nanocomposites in comparison with the pure chitosan nanoparticles.

### Evaluation of in vitro drug release

Investigating the DOX release from the carrier of chitosan nanoparticles and BSG/chitosan nanocomposites with weight percentages of different BSG was carried out in buffers at different pHs. For this purpose, the carriers were dispersed in buffers and stirred gently. 2 ml of the solution was extracted at specified times (0.5, 1, 2, 4, 6, 8, 24, 48 h and 4, 7, 14, 21, 28 days) and the amount of drug in the solution was measured by a UV–vis spectrometer at a wavelength of 480 nm. To maintain the volume of buffer, the release container was sealed to prevent solvent evaporation. Also, the same amount of the fresh buffer was replaced after each sampling.

### Evaluation of nano-carrier cytotoxicity

The cytotoxicity of the carriers synthesized without drug loading was investigated using the MTT assay in 2D and 3D culture on SKBR-3 breast cancer cell line (SKBR-3 cell line prepared from Pasteur Institute of Iran cell bank). For evaluation of 2D culture, monolayer cancer cells were prepared as follows: SKBR-3 cells cultured in DMEM medium at pH of 7.2–7.4 (containing FBS, penicillin, l-glutamine, and streptomycin) in a 37 °C incubator with 5% CO_2_. The cells grown up to 70–80% were dissociated by trypsin–EDTA^44^. Cultured monolayer cancer cells were treated with synthesized carriers (chitosan nanoparticles and BSG/chitosan nanocomposites with different wt% of BSG) for 24 h. Toxicity of carriers on the cells was determined using MTT assay by adding MTT solution (20 μL) to each well and incubating for 4 h to form formazan crystals followed by addition of DMSO (100 μl) and incubating again for 1 h to solubilize of formazan crystals and finally the absorbance of each well at 570 nm were obtained by a BioTek plate reader.

To evaluate carrier toxicity in 3D culture, micro-tumor tissue models from SKBR-3 cell spheroids were prepared. These spheroids of cancer cells were formed in a 3D printed-based micro-well array made of non-cell adherent hydrogel^45^. To meet this target, the scaffolds of agarose were steeped into culture media, followed by cell seeding with concentration of 500 × 103 cell/200 µL, and finally incubated within 4 days for spheroids formation. The spheroids formed were treated with nanocomposites and chitosan nanoparticles for 24 h, and their cytotoxicity was determined using the MTT assay. To each of the spheroid wells, a solution of MTT (600 μL) was added and incubated for 4 h to form formazan crystals, followed by the addition of DMSO (3 ml) and incubating again for 3 h to solubilize of formazan crystals, and finally, the absorbance of each well at 570 nm was obtained by a BioTek plate reader.

### Evaluation of drug release in 2D and 3D culture

To confirm the controlled and pH-sensitive release of DOX from BSG-chitosan nanocomposite 2wt% of BSG (optimal nanocomposite) and to compare the results of in vitro drug release with in vivo release, the effect of optimal nanocomposite treatment on cancer cells in 2D and 3D culture models were evaluated. Monolayer cells and cancerous spheroids for 2D and 3D culture were prepared from the SKBR3 cell line according to the method described in the evaluation of carrier cytotoxicity section. Cytotoxicity of DOX released from nanocomposite on monolayer and spheroid cancer cells at two pHs (4.5 and 7.4) and at four different times (1, 4, 6, 24, and 48 h) by MTT assay (as described in the evaluation of carrier cytotoxicity section) was measured.

### Statistical analysis

Quantitative results were expressed as mean ± SEM for the three experiments. Data were analyzed by one-way ANOVA, followed by Tukey’s multiple comparisons using GraphPad Prism 8 software. P values < 0.05 were considered as significant differences.

### Evaluation of release mechanism

To analyze the release of DOX from the carrier, release data were fitted using the zero-order model with formula (1), the first-order model with formula (2), the Higuchi model with formula (3), and the Korsmeyer-Peppas model with formula (4).

1 $${\mathrm{Q}}_{\mathrm{t}}:{\mathrm{K}}_{0}\mathrm{t}$$

2 $${\mathrm{logC}}_{\mathrm{t}}:-\frac{{\mathrm{K}}_{1}\mathrm{t}}{2.303}$$

3 $${\mathrm{Q}}_{\mathrm{t}}:{\mathrm{K}}_{\mathrm{H}}{\mathrm{t}}^{1/2}$$

4 $${\mathrm{Q}}_{\mathrm{t}}:{\mathrm{K}}_{\mathrm{KP}}{\mathrm{t}}^{\mathrm{n}}$$

The value of release exponent in the Korsmeyer-Peppas model describes the type of mechanism. If the release exponent value is less than 0.34, the release is under Fick’s diffusion law, while the release exponent value is more significant than 0.85 indicates release under case II transport (polymer relaxation). The release mechanism for release exponent value between 0.34 and 0.85 is a combination of Fick’s diffusion law and case II transport (anomalous)^46^.

## Results and discussion

**Figure 1:**
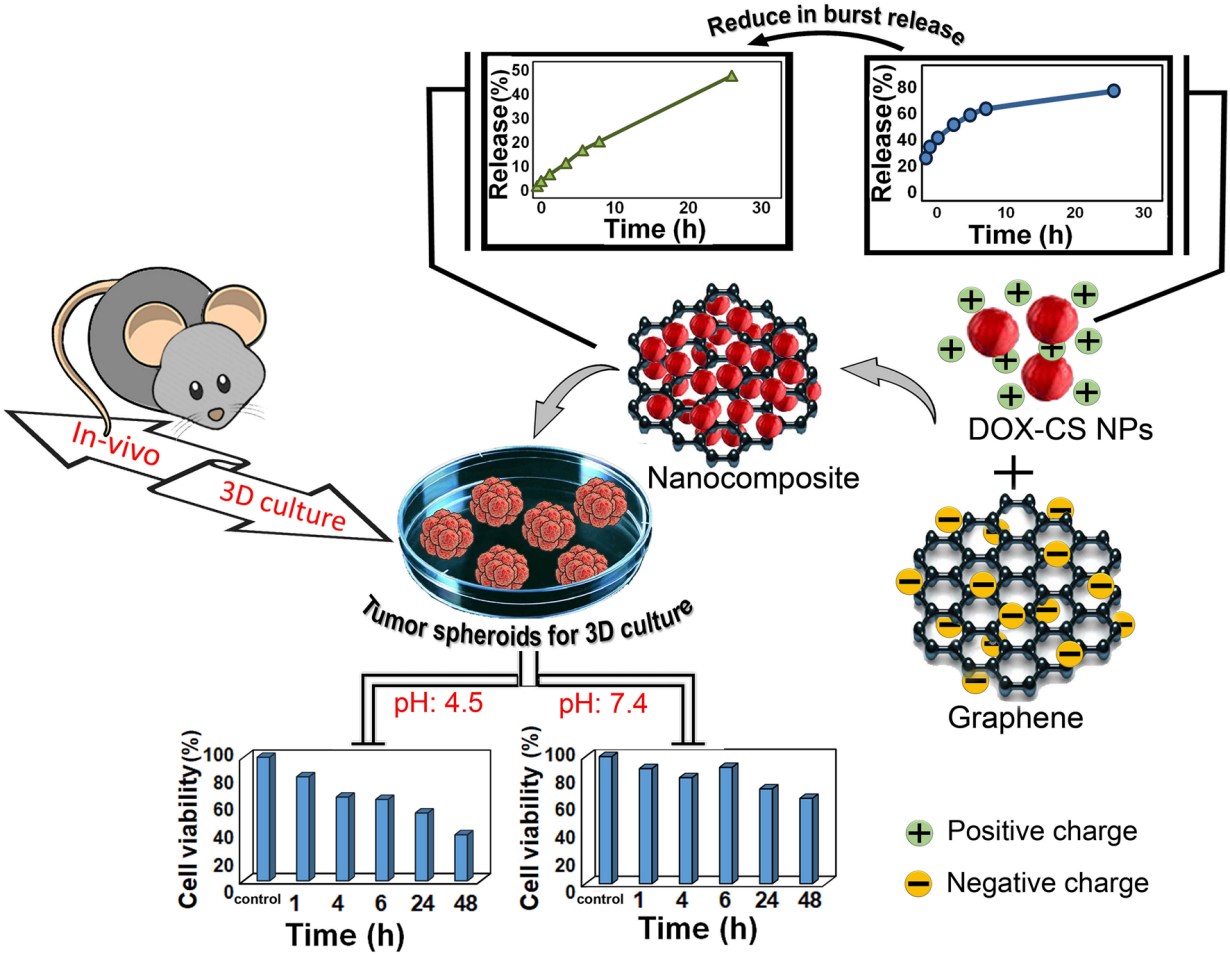
Schematic representation of the fabrication process of BSG/chitosan nanocomposites and comparison of release with chitosan nanoparticles and study of 3D culture.

In this work, as illustrated in Fig. 1 the BSG/chitosan nanocomposite was prepared by electrostatic interactions between the positive charge of chitosan nanoparticles and negative charge of BSG, which showed a more controlled and sustained release behavior compared to chitosan nanoparticles. Moreover, it was displayed that synthesized composite nanoparticles have a significant pH-sensitive drug release behavior in a 3D (spheroid) culture system.

In this regard, physicochemical and biological properties of the prepared nanocomposite as a potential smart drug carrier were characterized as follow.

### Physical and chemical characterization of the DOX-encapsulated chitosan nanoparticles

**Figure 2:**
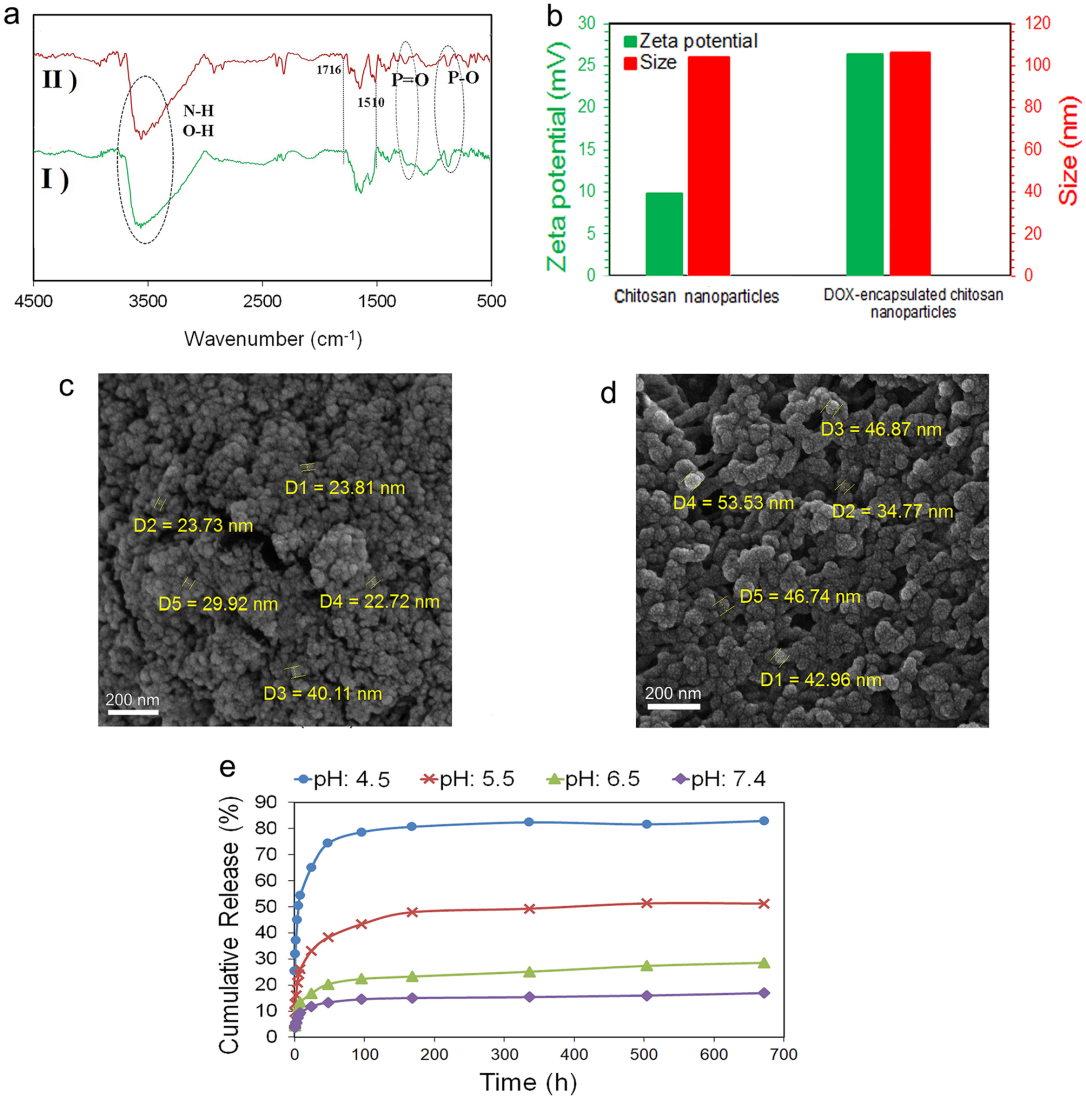
(**a**) FTIR spectra of (I) chitosan nanoparticles and (II) DOX-encapsulated chitosan nanoparticles, (**b**) the size and zeta potential values of chitosan nanoparticles and DOX-encapsulated chitosan nanoparticles dispersed in distilled water, (**c**) FESEM image of chitosan nanoparticles, (**d**) FESEM image of DOX-encapsulated chitosan nanoparticles, and (**e**) the release profile of DOX from chitosan nanoparticles at pHs 4.5, 5.5, 6.5 and 7.4 within 28 days.

The FTIR spectrum of chitosan nanoparticles and DOX-encapsulated chitosan nanoparticles are shown in Fig. 2a. In the chitosan nanoparticles IR spectrum, stretching vibration peaks of P=O and P–O at 1213 cm^−1^ and 870 cm^−1^, respectively, represent the formation of chitosan-tripolyphosphate nanoparticles^47^. In the IR spectra of the DOX-encapsulated chitosan nanoparticles, the appearance of 1716 cm^−1^ and 1510 cm^−1^ peaks are related to the vibration of the carbonyl ketone group and C–C vibration of the benzene ring in the doxorubicin structure and confirms the presence of the drug encapsulated inside the chitosan nanoparticles structure^48,49^.

The zeta potential and size of pure chitosan nanoparticles and chitosan nanoparticles encapsulated with DOX are shown in Fig. 2b. Pure chitosan nanoparticles have a positive zeta potential of + 10 mV due to the protonation of NH_2_ groups by acetic acid that are not neutralized by TPP anions^37,50^. The presence of NH_3_^+^ functional groups in the structure of DOX leads to increase in the surface charge of DOX-encapsulated chitosan nanoparticles to + 27 mV, compared to pure chitosan nanoparticles which confirms the encapsulation of DOX^51,52^. Also, chitosan nanoparticles and chitosan nanoparticles encapsulated with DOX show sizes of about 110 nm.

FESEM images in Fig. 2c,d show the morphology and size of synthesized chitosan nanoparticles and DOX-encapsulated chitosan nanoparticles which have a uniform morphology with regular spherical shapes. Chitosan nanoparticles are in the size of 20 to 40 nm, while DOX-encapsulated chitosan nanoparticles have a larger particle size of about 30–60 nm due to drug loading. The difference in size observation of the DLS method compared to the FESEM is attributed to the measurement mechanism of these two methods so that, DLS method measures the hydrodynamic diameter of the nanoparticles. Since chitosan nanoparticles swell in aqueous media, their hydrodynamic diameter is greater than their dry powder size, and hence a larger diameter was reported in the DLS method^37^.This particle size can fill the requirements with passive tumor targeting. Although various factors affect EPR and the cut-off size of permeable vascular varies from case to case, according to the literature, nanoparticles with a size of below 100 nm can easily pass through abnormal endothelial junctions of tumor blood vessels by utilizing the effect of EPR and create passive targeted drug delivery^28^.

The release profile of DOX from chitosan nanoparticles in buffer solutions with different pH values of 4.5, 5.5, 6.5, and 7.4 within 28 days is exposed in Fig. 2e. As it is clear, chitosan nanoparticles exhibited pH-sensitive release behavior, so that the release rate get faster by gradually changing the media pH from acidic to neutral. In the neutral environment with a pH of 7.4 within 28 days, the maximum drug release of 16.8% was acquired while, in the acidic environment with a pH of 4.5, 82.8% of the drug was released. The pH-sensitive behavior of chitosan nanoparticles with more release in the acidic environment is due to the presence of NH_2_ groups in the chitosan polymer chain. In acidic media, NH_2_ groups are protonated and converted to NH_3_^+^. The presence of NH_3_^+^ groups with positive charge leads to repulsion between the chitosan chains and consequently swelling of the nanoparticles. The more acidic the environment, the higher percentage of protonation, and consequently the greater repulsion between the chains occurs. This behavior results in more and faster drug release. This evidence represents the pH-dependent release of DOX through which the less unwanted DOX releases from the carrier in the normal tissue and thus does not cause any side effects compared to the conventional systemic delivery. The drug release curve showed that the DOX release profile of the chitosan nanoparticles samples follows 2 distinct behaviors, including a burst release up to 8 h, and a sustained release up to 672 h (28 days). The immediate burst release in acidic environments was more pronounced compared to the basic condition, so that, in the acidic environment with a pH of 4.5, more than 50% of the DOX payload was released in the first 8 h, as proof the burst release in the early hours.

### Physical and chemical characterization of BSG nanosheets

**Figure 3:**
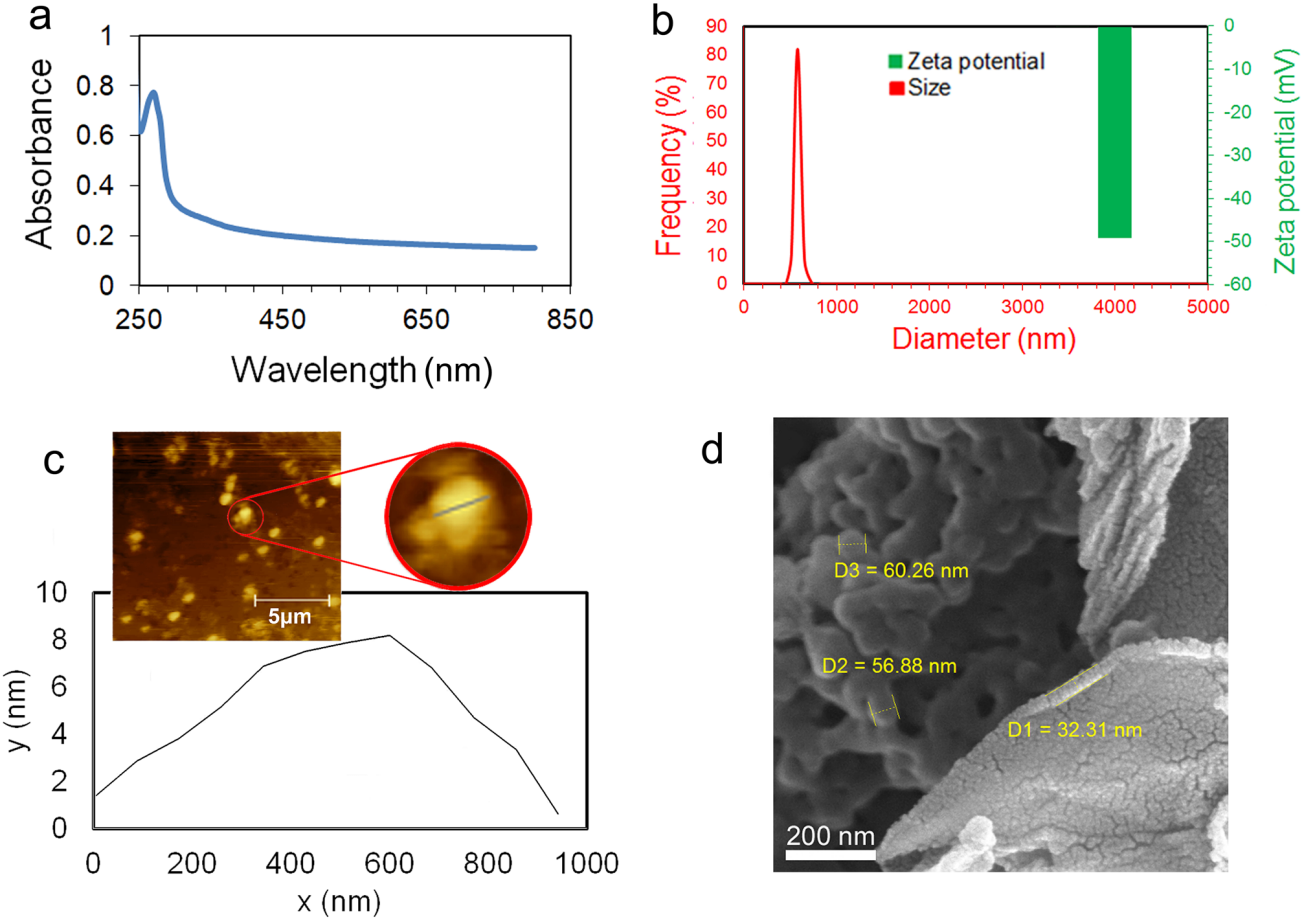
(**a**) The UV–vis spectrum of BSG, (**b**) the size distribution and zeta potential values of BSG dispersed by the ultrasonic probe in water at pH 10, (**c**) the AFM image and thickness profile of BSG, and (**d**) FESEM images of BSG/chitosan nanocomposite.

In the present work, the BSG nanosheet was synthesized through sonication of graphite in an aqueous solution of BSA. In this regard, the presence of dispersed BSG nanosheets in the aqueous solution of BSA was confirmed using UV–vis spectroscopy. Figure 3a exhibits the UV–vis spectrum of the synthesized BSG.

In order to obtain the graphene spectrum, the spectra of the BSG solution were recorded with the BSA blank solution. As shown, the absorbance peak appearing in the range of 270 nm, is the characteristic peak of BSG and is related to the π → π* transitions of the C–C aromatic ring^43^. Due to the presence of BSA protein in the structure of the synthesized graphene nanosheet, they are being expected to have different surface charges in water with various pH values^43,53,54^. In the case of the dispersion of BSG in acidic conditions, BSG will expose a positive surface charge^55^. In contrast, dispersion of BSG in the basic condition leads to the appearance of the negative surface charge due to the conversion of the COOH groups presented in the amino acid units of the protein to COO^−^ groups^55^. Formation of BSG/chitosan nanocomposite requires the electrostatic interaction of positive charge of DOX-encapsulated chitosan nanoparticles with negative surface charge BSG. As a result, for the synthesis of the nanocomposite, BSG was dispersed in water at a pH of about 10 to generate a negative zeta potential for BSG. Figure 3b shows the size distribution and zeta potential of the dispersed BSG in water at pH 10. BSG has a surface charge of − 50 mV and a size distribution in the range of 400–800 nm.

The AFM analysis was used to determine the thickness of the synthesized BSG nanosheets, (Fig. 3c). The profile of BSG nanosheets depicted a thickness of about 7 nm, attributed to the single-layer or few-layers BSG^56^.

### Physical and chemical characterization of BSG/chitosan nanocomposites

The FESEM images of BSG/chitosan nanocomposites in Fig. 3d show the microstructure of the electrostatically formed nanocomposite based on the electrostatic interaction between the positive charge of chitosan nanoparticles and the negative charge of BSG. Chitosan nanoparticles with positive charge were decorated on negatively charged BSG nanosheets, which showed the formation of BSG/chitosan nanocomposites.

**Figure 4:**
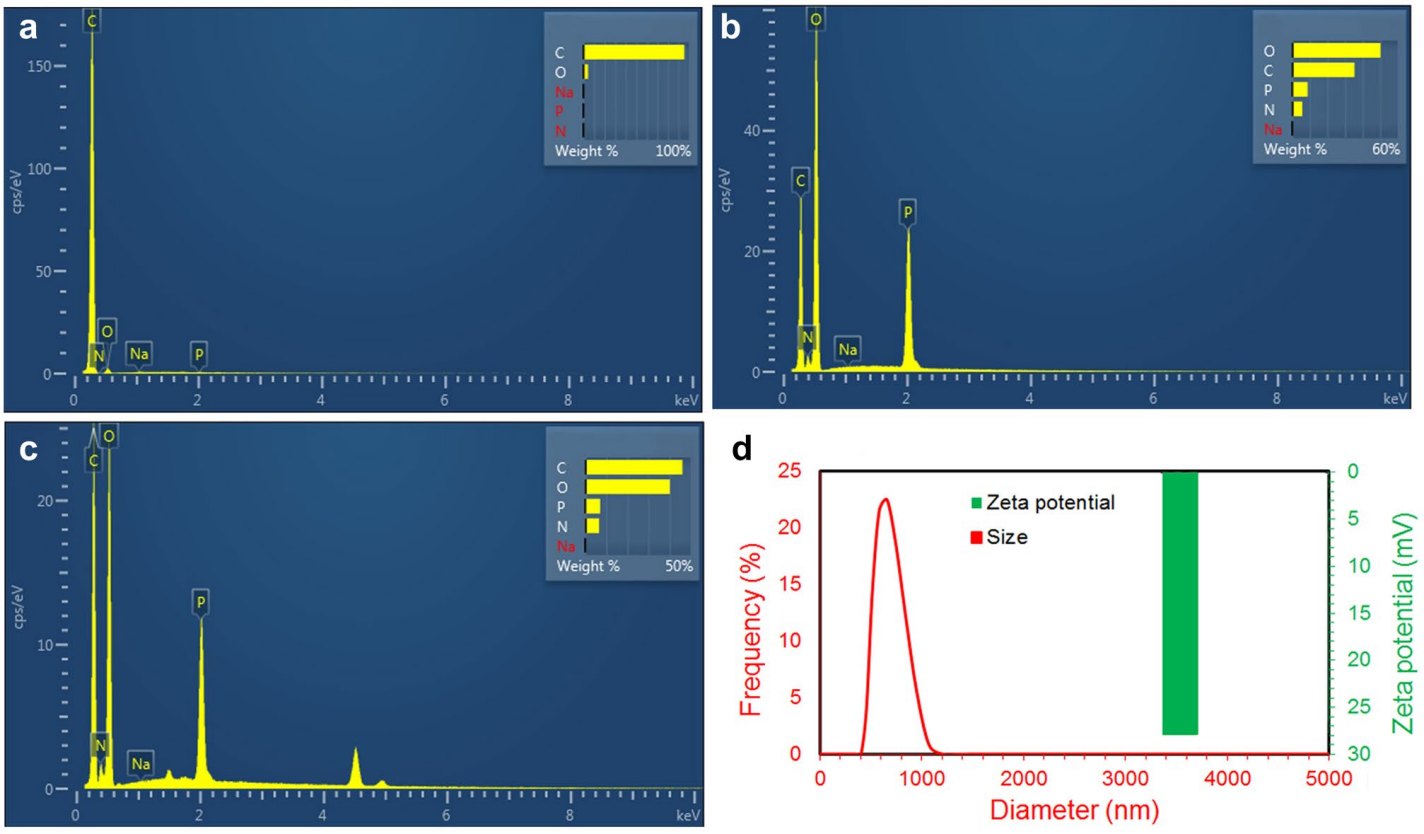
EDAX spectrum of (**a**) BSG nanosheets, (**b**) chitosan nanoparticles, (**c**) BSG/chitosan nanocomposites, and (**d**) the size distribution and zeta potential values of BSG/chitosan nanocomposites.

EDAX test was performed for chitosan nanoparticles, BSG nanosheets and BSG-chitosan nanocomposites (Fig. 4a–c). Figure 4a shows the EDAX spectrum of BSG nanosheets. The results exhibit the presence of a sharp peak for C, which is the main constituent of BSG nanosheets. In the EDAX spectrum of chitosan nanoparticles shown in Fig. 4b, the main elements are O, C, P, and N. The presence of peak of P is related to TPP, which was used to synthesize chitosan nanoparticles. Figure 4c shows the EDAX spectrum of the BSG-chitosan nanocomposites and exhibits the distribution of C, N, O, and P elements. The appearance of the peak of P in the BSG/chitosan nanocomposites spectrum proves the presence of chitosan nanoparticles. Also, increasing the wt% of C element in the BSG/chitosan nanocomposites spectrum, compared to the spectrum of chitosan nanoparticles proves the presence of BSG nanosheets. These results confirm the formation of BSG/chitosan nanocomposites from chitosan nanoparticles and BSG nanosheets.

Figure 4d shows the zeta potential and size distribution of BSG/chitosan nanocomposites. The size of BSG/chitosan nanocomposites with a surface charge of about + 28 mV, is in the range of 400 to 1000 nm. Zeta potential is one of the important characteristics of carriers and affects their effectiveness. This level of surface charge ensures the stability of the synthesized nanocomposite in suspension.

### Encapsulation efficiency and loading capacity

In order to have a smart drug delivery for the sustained drug release in the tumor microenvironment, the nano-carriers system should be able to encapsulate the large volume of the drug payload. In this regard, the drug encapsulation efficiency of the drug delivery depot systems is considered as one of their most prominent characteristics. The encapsulation efficiency of the DOX for chitosan nanoparticles was calculated using Eq. (5).

5 $$\% \; \text{Encapsulation} \; \text{efficiency}=\frac{\text{Mass} \;  \text{of}\;  \text{DOX}\; \text{in} \;\text{nanoparticles}}{\text{Mass} \; \text{of} \; \text{DOX} \; \text{used} \; \text{for} \; \text{nanoparticles}}\times 100$$

where the mass of DOX used for the nanoparticles is the same as the quantity of DOX added initially during the preparation of the nanoparticles. To determine the mass of DOX in nanoparticles, the mass of unencapsulated DOX (free drug) was measured by determining the concentration of drug in the supernatant collected after the synthesis of nanoparticles using a UV–vis spectrometer at 480 nm and from the mass of used initial drug was subtracted. According to this equation, the encapsulation efficiency of DOX for chitosan nanoparticles was reported around 80%. Additionally, the loading capacity of DOX in the nanocomposite structure was also calculated according to Eq. (6), and it was reported to be around 4% for BSG/chitosan nanocomposite.

6 $$\% \; \text{Loading} \; \text{capacity}=\frac{\text{Mass} \; \text{ of} \; \text{DOX} \; \text{ in} \; \text{nanocomposite}}{\text{Mass} \; \text{of} \; \text{nanocomposite }}\times 100$$

In this equation, the mass of DOX used for nanocomposite and the mass of DOX in nanocomposite were calculated as in Eq. (5).

### In vitro drug release study

**Figure 5:**
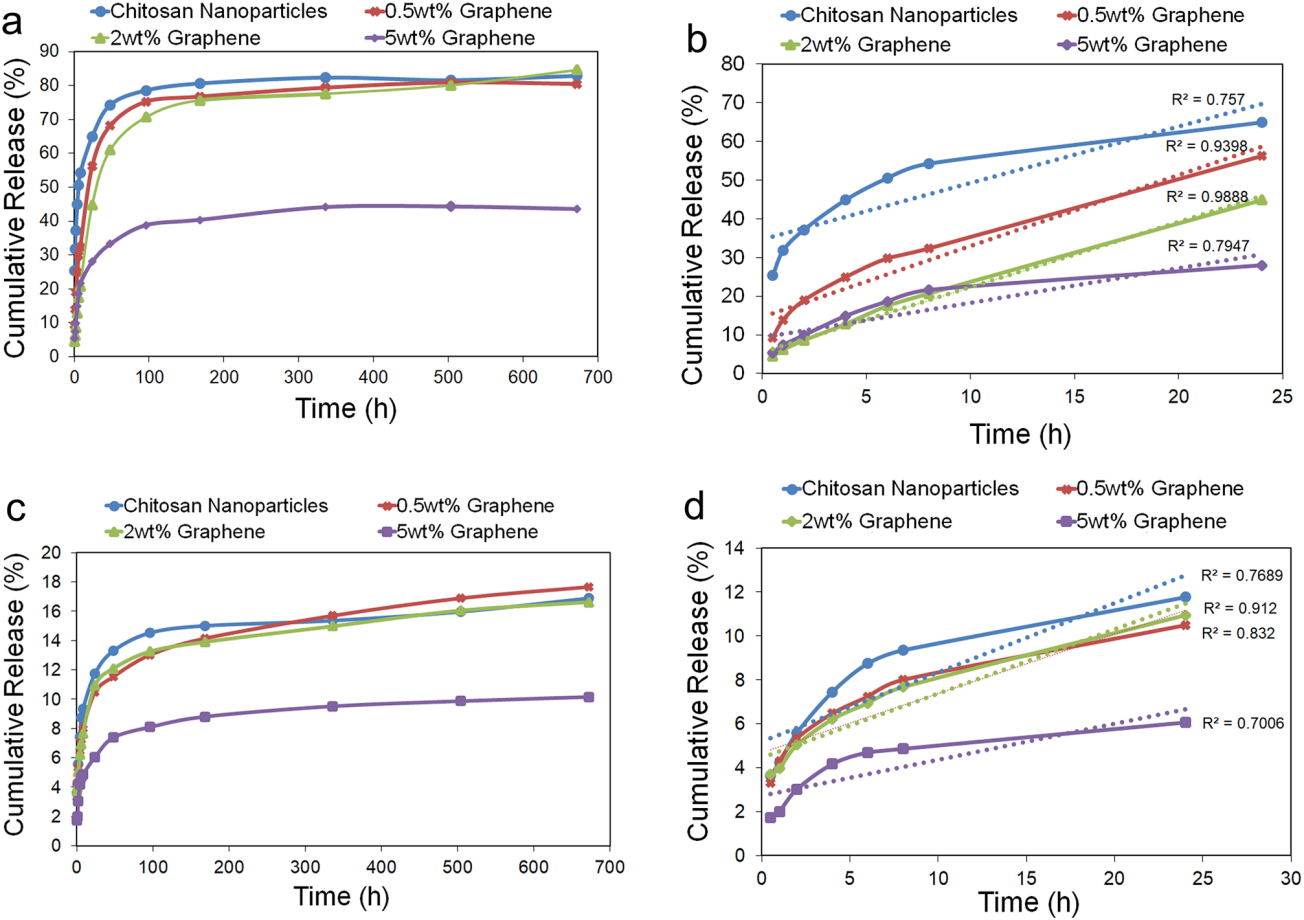
The release profile of DOX from the BSG/chitosan nanocomposite of 0.5, 2, and 5wt% BSG and its comparison with the curve of chitosan nanoparticles (**a**) at pH 4.5 within 28 days, (**b**) at pH 4.5 within the first 24 h. (**c**) at pH 7.4 within 28 days, (**d**) at pH 7.4 within the first 24 h.

The release behavior of DOX from three compositions of the BSG/chitosan nanocomposites containing 0.5, 2, and 5wt% BSG was investigated and compared with the release curve of DOX from chitosan nanoparticles at pH 4.5 (Fig. 5a,b). It has been shown that nanocomposites of 0.5 and 2wt% BSG have the same release behavior as chitosan nanoparticles so that an accumulative DOX release of around 83% was reported on day 28. However, at the primary phase time of the release, nanocomposite samples showed a more controlled and slower release than chitosan nanoparticles. On the other hand, the nanocomposite sample of 5wt% BSG only released 43.5% of its DOX content within 28 days. The controlled release behavior of the nanocomposites of 0.5 and 2wt% BSG was due to the presence of BSG nanosheets. The presence of more BSG nanosheets in the structure of the nanocomposite (5wt% BSG) reduced the release rate of the drug content. Figure 5b shows the drug release profile within the first 24 h at pH 4.5. As can be seen, accumulative DOX release from the chitosan nanoparticles carriers in the first 8 h of the release experiment is around 54%, while this amount in the nanocomposite of 0.5 and 2wt% BSG decreased to 32.3% and 20.6%, respectively. The nanocomposite of 5wt% BSG exhibited a release behavior similar to the nanocomposite of 2wt% at first 8 h, but after 8 h, its release rate significantly decreased, so that after 28 days, the total amount of released drug did not reach about 83%. The nanocomposite of 2wt% BSG with the highest linear regression coefficient showed the most uniform release within 24 h.

DOX release experiments of BSG/chitosan nanocomposite samples were also conducted at pH 7.4, and the results compared with the profile of chitosan nanoparticles (Fig. 5c,d). According to the release behavior of three nanocomposites in a neutral environment with a pH of 7.4, the nanocomposites of 0.5 and 2wt% BSG again showed a release behavior similar to chitosan nanoparticles as discussed for the pH 4.5 experiment. However, the accumulative release of DOX within 28 days was about 17%. The greater amount of BSG in the nanocomposite structure (5wt% BSG) similarly led to a reduction in the total drug released rate at the pH of 7.4, so that only 10% of the drug was released within 28 days. While chitosan nanoparticles showed an accumulative release of around 17% on day 28. Figure 5d shows drug release within the first 24 h at pH 7.4. Release amount of about 10% of DOX from chitosan nanoparticles up to 8 h decreased to about 7% for two nanocomposites of 0.5 and 2wt% BSG. The nanocomposite of 2wt% BSG showed the most uniform release with the highest linear regression coefficient at pH 7.4 within 24 h.

This different release behavior of nanocomposite and chitosan nanoparticles in different pHs due to the presence of BSG nanosheets. The more controlled release of the BSG/chitosan nanocomposite compared to chitosan nanoparticles in both pH is due to interactions between DOX and BSG nanosheets via π–π stacking. The release behavior of nanocomposites at different pHs can also be attributed to the hydrogen bond between BSG nanosheets and the DOX. This hydrogen bond is more prominent in the neutral environment, leading to the amount of less drug release. In acidic conditions, the carboxylic acid groups in the amino acid units of BSG are protonated, resulting in partial dissociation of the hydrogen bond between the BSG nanosheets and the drug. Hence, the amount of more drug is released from the nanocomposites. The difference in the release of nanocomposite containing different amounts of BSG is due to different degrees of interactions (π–π stacking and hydrogen bonding) between BSG nanosheets and the DOX.

Table 1 lists the pH-sensitive behavior of several chitosan-based nano-carriers. As an example, Anirudhan et al. synthesized graphene oxide-based functionalized chitosan polyelectrolyte nanocomposite as a pH-sensitive and targeted drug delivery system. They showed that the release rate of DOX from this nanocomposite was about 86% within 24 h in an acidic medium (pH: 5.3)^57^. In the study, Rahimi et al. synthesized rod-like chitosan-quinoline nanoparticles as pH-sensitive nano-carriers and investigated the release of quercetin at pH 5.8 and 7.4 for 150 h. Chitosan nanoparticles cross-linked with 2-chloro-3-formylquinoline (CFQ) showed a release rate of 65% in a neutral medium. These nanoparticles also showed burst release, which resulted in the release of about 56% and 78% of the drug at pH 7.4 and 5.8 in the first 8 h, respectively^58^. In another effort, Adimoolam et al. synthesized chitosan functionalized Fe_3_O_4_ nanoparticles using a simple hydrolysis method. Although these nanoparticles exhibited good pH-sensitive behavior with 90% and 10% doxorubicin release at pH 4.5 and 7.4, respectively, they released this amount within 8 h and did not provide sustained and prolonged release^59^.

**Table 1:** Maximum drug release in acidic and neutral medium and release time of some pH-sensitive nano-carriers.

Carrier type	Duration of release (h)	Maximum drug release in acidic medium (%)	Maximum drug release in neutral medium (%)	References
FA-CS-g-P(IA/AA))/AGO polyelectrolyte nanocomposite	48	86	45	^57^
Chitosan–quinolone nanoparticles crosslinked with CFQ	150	95	65	^58^
Chitosan nanoparticles prepared using SAA-HCM	48	80	20	^60^
Estrone-modified glycol chitosan nanoparticles	48	65	50	^61^
Chitosan nanoparticles containing AGS	24	94	57	^62^
Chitosan functionalized Fe_3_O_4_ nanoparticles	8	90	10	^59^

Therefore, the nanocomposite, while showing pH-sensitive behavior like chitosan nanoparticles, has a slower release. it can be interpreted that in acidic media, protonation of NH_2_ chitosan groups will still lead to pH-sensitive effects, but due to the presence of graphene plates in the carrier, the drug released from the nanoparticles interacts with the graphene plates. The interaction between the aromatic ring of graphene and DOX results in extremely controlled, stable, and long-lasting release.

### Cytotoxicity analysis of the DOX-encapsulated nanocomposite

**Figure 6:**
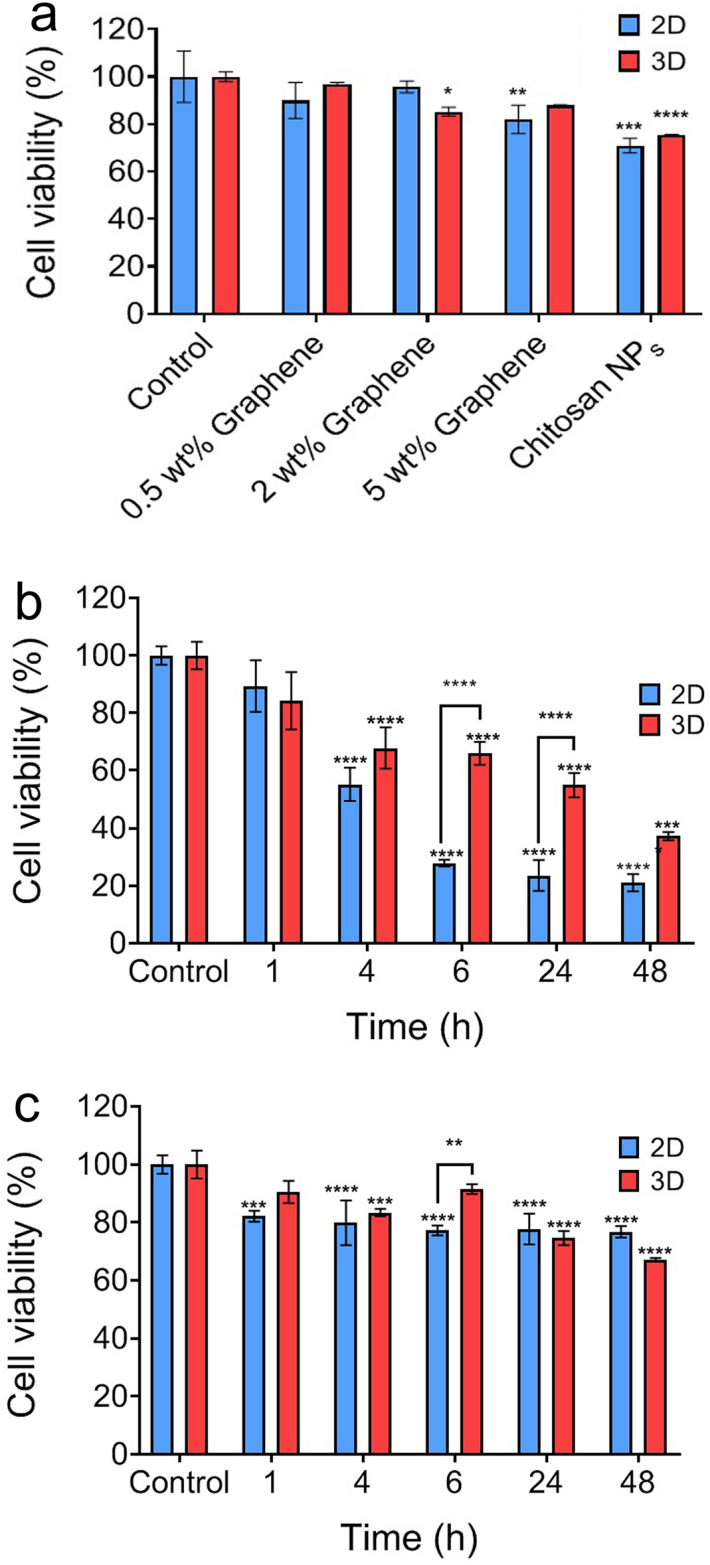
(**a**) Cytotoxicity of drug-free graphene/chitosan nanocomposites with 0.5, 2, and 5wt% of graphene and drug-free chitosan nanoparticles on SKBR-3 cell line in 2D and 3D cultures models using MTT assay, (**b**) cell viability of SKBR-3 cancer cells with DOX released after treatment with the graphene/chitosan nanocomposite with 2wt% of graphene in 2D and 3D cultures at pH 4.5 and (**c**) cell viability of SKBR-3 cancer cells with DOX released after treatment with the graphene/chitosan nanocomposite with 2wt% of graphene in 2D and 3D cultures at pH 7.4. Values are mean ± SEM (n = 3). Significant difference (P < 0.05) was marked with * (p < 0.0001: ****, p < 0.001: ***, p < 0.01: **, p < 0.05: *).

To evaluate the cytotoxicity of the synthesized carriers, the cell viability of SKBR-3 cells treated with nanocomposites and chitosan nanoparticles in 2D and 3D culture models was measured by MTT assay for 24 h. As shown in Fig. 6a, the induced toxicity of the chitosan nanoparticles compared to the control sample seems to be related to the zeta potential of the chitosan at culture medium pH resulting in a slightly acidic environment and subsequently cell death. Although carbon-based materials exhibit dose-dependent toxicity, the addition of BSG to chitosan nanoparticles not only did not cause carrier toxicity but also increased cell viability. BSG was synthesized using an utterly green method, and in the process of its preparation, no toxic substance or solvent was used, and hence it showed no cytotoxicity. The increase in cell viability can also be attributed to the increased stability and resistance of BSG/chitosan nanocomposites against environmental changes. As a result, BSG/chitosan nanocomposites showed no significant toxicity, so that in both 2D and 3D culture conditions, the cell viability was above 80% compared to the control sample.

Figure 6b,c demonstrate the effect of DOX payload on cytotoxicity of the 2D and 3D cultured cells. It is blatant in Fig. 6b,c, that in both 2D and 3D culture platforms, an acidic pH environment could stimulate greater drug release in each time interval. It is demonstrated that after 48 h of release study, 76% of the SKBR-3 cells were viable at pH 7.4, while this value was decreased to 20% at pH 4.5. These data were compatible with accumulative drug release data in Fig. 5, which showed the pH sensitivity of the BSG/chitosan nanocomposite. The 10–15% decrease in cell viability after 1 h, and also no significant difference compared to the control group in both 2D and 3D culture models at pH 4.5, confirmed the successful reduction of burst release and acquiring controlled release manner compared to chitosan nanoparticles. Releasing about 30% of the drug content from chitosan nanoparticles after 1 h, which was reduced to about 5% from BSG/chitosan nanocomposite with 2wt% of BSG (As shown in Fig. 5b), could lead to the sudden decrease in cell viability that could cause undesirable side effects.

By comparing 2D and 3D cell culture platforms, cell viability of the tumor spheroids was greater than the cell monolayer cultured at both acidic and neutral pHs. This phenomenon shows the importance of 3D culture study in developing new drug delivery systems so that these tumor spheroids can better recapitulate the biological properties of the whole tumor in toxicity studies than the typical 2D cultured cells. Limited drug diffusion into the core of SKBR-3 spheroids is one of the main reasons for the lower induced toxicity for the tumor spheroids. Spheroid forming is followed by ECM secretion, cell infusion, and hence dense spheroid core formation. This kind of cell–cell contact and ECM secretion play a barrier role for facile drug diffusion and subsequently cell death. 3D cell culture by creating an environment for cell growth in 3D not only lacks the limitations of 2D culture and the complexities of the animal model but also provides more similarity to physiological conditions with a 3D reconstruction of in vivo environment. As a result, 3D culture systems as a more complex in vitro model can be used for biological research instead of in vivo models.

### Drug release mechanism study

The release mechanism of the drug from the chitosan nanoparticles was studied using zero-order, first-order, Higuchi, and Korsmeyer-Peppas models. The correlation coefficient (R^2^) and release exponent (n) obtained for chitosan nanoparticles in four conditions with the pHs equal to 4.5, 5.5, 6.5, and 7.4 are reported in Table 2. Concerning the amount of the correlation coefficient, it can be concluded that the release mechanism of DOX from chitosan nanoparticles is followed by the Korsmeyer-Peppas model in all four pHs. Also, the release exponent value of the Korsmeyer-Peppas model was calculated and according to n ≤ 0.34, it showed that the mechanism of release was under Fick’s diffusion law at all pH values.

**Table 2:** Values of the correlation coefficient and release exponent obtained for mathematical models applied to DOX release from chitosan nanoparticles.

	Zero-order	First-order	Higuchi	Korsmeyer-Peppas	
	R^2^	R^2^	R^2^	R^2^	n
pH: 4.5	0.7570	0.8469	0.9243	0.9878	0.2472
pH: 5.5	0.7788	0.8152	0.9378	0.9862	0.3267
pH: 6.5	0.7847	0.7996	0.9367	0.9787	0.3773
pH: 7.4	0.7689	0.7790	0.9306	0.9814	0.3267

The results of the DOX release from the nanocomposite of 2wt% BSG (as optimal composition) at two pH values of 4.5 and 7.4 were evaluated by zero-order, first-order, Higuchi, and Korsmeyer-Peppas models. The outcome of the correlation coefficient of zero-order, first-order, Higuchi, Korsmeyer-Peppas models and release exponent value of the Peppas model for the BSG/chitosan nanocomposite of 2wt% BSG in two pH conditions of 4.5 and 7.4 are reported in Table 3. In the environment with a pH of 7.4, the highest correlation coefficient was obtained for the Higuchi model. The Higuchi model shows that the kinetics follow t^1/2^, so we can interpret that controlled diffusion is the main mechanism of drug release^63^. The highest correlation coefficient was obtained in the environment with a pH of 4.5 for the first-order model. The first-order mechanism partly reflects the reservoir-type delivery system^64^. The reservoir-type system has an inert coating that acts as a rate-controlling membrane^65^. It can be concluded that graphene played the role of this inert coating.

**Table 3:** Values of the correlation coefficient and release exponent obtained for mathematical models applied to DOX release from the BSG/chitosan nanocomposite of 2wt% BSG.

	Zero-order	First-order	Higuchi	Korsmeyer-Peppas	
	R^2^	R^2^	R^2^	R^2^	n
pH: 4.5	0.9888	0.9989	0.9818	0.9916	0.5973
pH: 7.4	0.9120	0.9192	0.9945	0.9870	0.2890

Analysis of the release exponent value for the nanocomposite of 2wt% BSG in two media with the pH values of 4.5 and 7.4 showed that at pH 7.4, DOX was released from the nanocomposite followed by Fickian diffusion mechanism, while at pH 4.5, it was released followed by a combination of Fick’s diffusion law and Case II transport.

The addition of BSG nanosheets to the chitosan nanoparticles led to a change in the release mechanism from Fick’s diffusion to the diffusion and case II mechanisms in acidic pH. Diffusion occurs when the relaxation time is infinite or zero^66^. While appropriate polymer relaxation does not make diffusion the only mechanism of release, it provides relatively sustain release with a time close to zero-order release^67^. The change of R^2^ for the zero-order mechanism by adding BSG to the chitosan nanoparticles from 0.757 to 0.988 confirms this at pH 4.5. The prolonged and uniform release of the nanocomposite was due to the presence of graphene, which in addition to being able to π-π interact with DOX, provides the desired stability and mechanical properties of the nanocomposite.

## Conclusion

Here, the BSG/chitosan nanocomposites were prepared in three wt% of BSG (0.5, 2, and 5). To investigate the cytotoxicity, the effect of these nanocomposites on SKBR-3 cells was investigated in 24 h. All three nanocomposites exhibited less toxicity than chitosan nanoparticles. The release profile of DOX was obtained from three nanocomposites at pHs 7.4 and 4.5 for 28 days. The results showed that the BSG/chitosan nanocomposite presented a reduction of burst release compared to chitosan nanoparticles. The BSG/chitosan nanocomposite of 2wt% BSG with the most uniform release toward time and release of 84%, was selected as the optimal nanocomposite. The release mechanism study indicated that the release of DOX from chitosan nanoparticles followed the Korsmeyer-Peppas model at all pHs. The presence of BSG led to a change in the release mechanism at pH 4.5, to the first-order model and at pH 7.4, to the Higuchi model. Finally, the optimal nanocomposite treatment (2wt% BSG) was established on SKBR-3 cells in 2D and 3D culture models at the pHs 4.5 and 7.4 for 48 h, which the results of 3D culture were consistent with the results of in vitro release.
